# Effects of Different Monochromatic Light Combinations on Cecal Microbiota Composition and Cecal Tonsil T Lymphocyte Proliferation

**DOI:** 10.3389/fimmu.2022.849780

**Published:** 2022-07-12

**Authors:** Yijia Zhang, Zixu Wang, Yulan Dong, Jing Cao, Yaoxing Chen

**Affiliations:** Laboratory of Anatomy of Domestic Animals, College of Animal Medicine, China Agricultural University, Beijing, China

**Keywords:** monochromatic light combination, T lymphocyte, intestinal microbiota, metabolomic, cecal tonsil

## Abstract

Emerging data demonstrated that the gut microbiota plays an important role in protecting the integrity of the epithelial barrier, forming a mucosal immune system, and maintaining intestinal homeostasis through its metabolites. However, the intestinal microbiota community can be affected by environmental factors, such as litter, photoperiod, or temperature. Thus, we investigated the effect of different monochromatic light combinations on cecal microbiota composition as well as explored the molecular mechanism by how the external light color information mediate cecal tonsil T lymphocyte proliferation. In this study, a total of 160 chicks were exposed to monochromatic light [red (R), green (G), blue (B), or white (W) light] or green and blue monochromatic light combination (G→B) from P0 to P42. The 16S rRNA microbial sequencing results showed that the richness and diversity of the cecum microbiota and the abundance of *Faecalibacterium* and *Butyricicoccus* were significantly increased in the G→B. With consistency in the upregulation of antioxidant enzyme ability and downregulation of pro-inflammation levels in the cecum, we observed an increase in the number of goblet cells, secretory IgA+ cells, tight junction protein (occludin, ZO-1, and claudin-1) and MUC-2 expression in the cecum of the G→B. The metabolomics analysis revealed that the relative abundance of metabolites related to butyrate was significantly increased in G→B. In an *in vitro* experiment, we found that butyrate could effectively induce T lymphocyte proliferation and cyclin D1 protein expression. However, these butyrate responses were abrogated by HDAC3 agonists, STAT3 antagonists, or mTOR antagonists but were mimicked by GPR43 agonists or HDAC3 antagonists. Thus, we suggested that G→B can indirectly affect the composition of cecal microbiota as well as increase the relative abundance of *Faecalibacterium* and *Butyricicoccus* and butyrate production by reducing the level of oxidative stress in the cecum. Exogenous butyrate could promote the T lymphocyte proliferation of cecal tonsil by activating the GPR43/HDAC3/p-STAT3/mTOR pathways.

## Introduction

It has been reported that heritability only accounts for 5–50% of factors affecting poultry productivity performance, while 50–95% depends on environmental conditions (1). Light, as an important environmental factor, has significant impacts on chick behavior, production performance, and health status. Therefore, artificial lighting is widely used in modern chicken industry to promote chicken production performance and improve breeding efficiency. Chicks need to take in a large amount of foreign nutrients every day to meet their own growth and development, so the intestinal tract is always at risk of invasion by harmful pathogens. In poultry, there are 10^10^ bacterial colony-forming units per gram of digestive tract and approximately 1,000 different species harbored in the cecum (2). These diverse microorganisms play important roles in nutrient exchange, immune system regulation, and pathogen defense (3). At the phylum level, the *Firmicutes* and the *Bacteroidetes* are the two dominant bacteria in the cecum, with lesser contributions from other phyla (notably *Proteobacteria* and *Actinobacteria*). At the genus level, the most abundant groups in the chicken cecum were found to be *Ruminococcus*, *Bacteroides*, *Clostridium*, and *Eubacterium* (4). Although the dominant bacteria in the cecum have been widely recognized, the composition of microorganisms in the cecum can be influenced by a variety of environmental factors, such as temperature, location, litter, and housing (5). Chicks have developed visual systems, and they are very sensitive to light stimulation of the external environment. Recent studies have demonstrated that photoperiod could change the cecal microbial communities in chicks (6). However, apart from photoperiod and light intensity, the different wavelengths of light also have profound effects on the behavior, growth, and health state of chicks. A previous study found that 560-nm green light promotes muscle growth (7), increases satellite cell mitotic activity (8), and alleviates oxidative stress (9) during the early stage [post-hatching (P) 0–P26)], and 480-nm blue light is more effective during the later stage (P27–P42). Overall, these results indicated that post-hatching 26 is a critical point between the early and later stages of growth. Therefore, a combination of green and blue monochromatic light (G→B) at P26 was designed to determine whether a combination of monochromatic lights could improve the productive performance and enhance the immune function of the chicks. Our lab previously found that the chicks which were raised under G→B have better production performance and can produce more antibodies (10). In addition, melatonin plays an important role in the process of converting environmental light stimulation into internal molecular signals. However, the effect of different monochromatic light combinations on chick cecum microbiota composition and structure is unclear.

Consider that the gut is exposed to a large number of external substances from the outside world and may be threatened by harmful pathogens at any time. Thus, the mucosal immune system is crucial in protecting the gut from pathogen invasion and maintaining intestinal homeostasis. The intestinal mucosa immunity system contains intestinal epithelium mucosal barrier, intestinal-associated lymphoid tissues (GALT), and secretory IgA (sIgA) plasma cells. The mucosal barrier systems were composed of physical barrier and chemical barrier. The cecum has a less developed chemical barrier than the small intestine because there is almost no distribution of Panth cells (11). In contrast, there exist well-developed physical barriers in the cecum that separate the intestinal epithelial layer and commensal microorganisms. The physical barrier in the cecum is made up of three parts. The first physical barrier is the mucous layer, which consisted of goblet cell-secreted MUC-2 protein (12). The second physical barrier is a glycocalyx, including MUC-1, MUC-13, and MUC-17 (13). The third physical barrier is tight and adhesion junctions including claudins, occludin, and ZO-1 (14). In chickens, the cecal tonsil is the major lymphoid tissue within the GALT, and the induction of IgA also takes place in the germinal centers of the cecal tonsil. In addition, the cecal tonsil also contains a large number of T lymphocytes. On the one hand, T lymphocytes can secrete cytokines, maintain intestinal immune homeostasis, and promote epithelial cells to secrete antibacterial peptides; on the other hand, they can also activate B lymphocytes to differentiate into plasma cells and secrete IgA. In conclusion, the intestinal mucosal immune system plays an important role in maintaining intestinal homeostasis and preventing the invasion of harmful pathogens. Thus, an investigation into whether different monochromatic light combinations affect chicken intestinal mucosal immune function is still required.

As it is well known that intestinal microbes play a key role in regulating immune capacity and the metabolism of the body, gut microbes perform these functions mainly through its metabolites. A recent review detailed how metabolites derived from gut microbes regulate the metabolism and function of immune cells (15)—for example, short-chain fatty acids (SCFAs), particularly butyrate, can not only provide energy for intestinal epithelial cells and accelerate their proliferation and circulation cycle but also regulate the function of innate myeloid cells in the intestinal tract and promote the repair of mucosal damage (16). In addition, tryptophan and its derivatives also play an important role in the maintenance of epithelial barrier integrity and the recovery of intestinal epithelial barrier injury. Thus, one question arises: How do the different monochromatic light combinations affect the mucosal immune function?

Here we specifically discuss the cecal microbiota composition under different monochromatic light combinations in chicken as well as explored the intracellular molecular mechanism of intestinal microbial influence on cecal tonsil T lymphocyte proliferation *in vitro*. We considered that our results will provide another lighting solution for the rational use of artificial light to reduce intestinal stress levels, enhance gut health condition, and promote the growth and development of chicks.

## Materials and Methods

### Animals and Treatments

All experiments were carried out under the approval of the Animal Welfare and Ethics Committee of China Agricultural University. The permit number was CAU 20171114-2.

A total of 160 post-hatching day P0 Arbor Acre male chicks (Beijing Hua du Breeding Co., Beijing, China) were randomly divided into four light treatment groups (*n* = 40), including white light group (400 to 700 nm, WW), red light group (660 nm, RR), green light group (560 nm, GG), and blue light group (480 nm, BB) by a LED system. In addition, the light regime was 23:1 light/dark (light on at 08:00 and off at 23:00). Therefore, on P26 at 23:00, after the lights were turned off, 20 chickens from the green light were transferred to the blue light. The remaining chickens were maintained under the original light color. Therefore, the four light control groups (before P27) were changed into five light groups until P42. The five light groups were as follows: WW, RR, GG, BB, and G→B [for more details, refer to a previous description (17)]. The light parameters are shown in **Table 1**.

**Table 1:** Light parameters.

	WW	RR	GG	BB	G→B
Light wavelength, nm (1–26 days)	400–700	660	560	480	560
Light wavelength, nm (27–42 days)	400–700	660	560	480	480
Light intensity (W/m^2^)	0.19	0.19	0.19	0.19	0.19
Photoperiod [(Llight:/Ddark])	23:1	23:1	23:1	23:1	23:1

### Sampling

**Figure 1:**
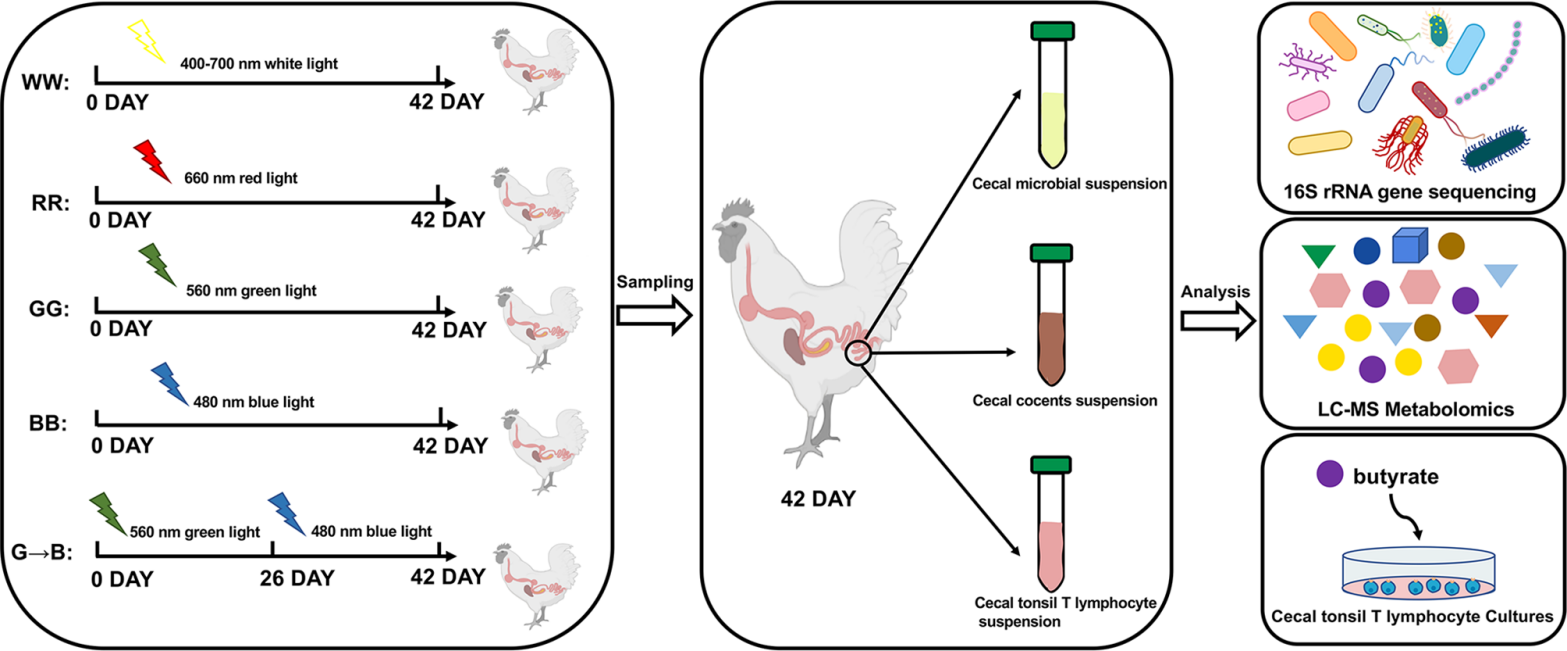
An overview of the experiment design and sample collection. A total of 160 post-hatching day P0 Arbor Acre male chicks were randomly divided into four light treatment groups (*n* = 40), including white light group (400 to 700 nm, WW), red light group (660 nm, RR), green light group (560 nm, GG), and blue light group (480 nm, BB) by a LED system. On P26 at 23:00, after the lights were turned off, 20 chickens from the green light treatment were transferred to the blue light treatment. The five light groups were as follows: WW, RR, GG, BB, and G→B. At P42, all chicks were euthanized, and then their cecum tissue and cecum content were harvested for microbial sequencing and metabolomics profiling. In addition, T lymphocyte was obtained aseptically from cecal tonsil in G→B. Then, a single-cell suspension was prepared with RPMI 1640 medium and then stimulated with butyrate to verify if exogenous butyrate promoted the proliferation of T lymphocytes *in vitro*.

At P42, all chicks were euthanized, and then their cecum tissue and cecum content were harvested. Four chicks were randomly selected from WW, RR, GG, BB, and G→B, and the cecum content was collected for microbial sequencing. In addition, six chicks in the WW, RR, GG, BB, and G→B were used for inflammatory factors and antioxidant capacity assay. The fresh cecum content was collected for volatile fatty acid (VFA) analysis. Five other chicks from each of the remaining chicks in the WW, RR, GG, BB, and G→B were selected, and the whole cecum was collected and divided into 2 parts, which were used for immunohistochemistry and Western blot analysis (for more details on the sampling and analysis protocol, refer to **Figure 1**).

### Lymphocyte Proliferation Assay

T lymphocyte was obtained aseptically from cecal tonsil and cultured in RPMI 1640 medium. Then, T lymphocyte was stimulated with ConA (20 μg/ml, Sigma, St. Louis, MO, USA) + butyrate (0.5 mM, Sigma, St. Louis, MO, USA) and incubated at 37°C with 5% CO_2_ for 44 h. The proliferation level of T lymphocyte was determined by a methyl thiazolyl tetrazolium assay. We used the stimulation index (SI) to express the proliferative activity of T lymphocyte: SI = OD570 (stimulated cells)/OD570 (unstimulated cells).

In addition, cecal tonsil T lymphocytes were prepared with either 20 μM 4-CMTB (a GPR43 agonist, MCE, New Jersey, USA), 10 nM TSA (an HDAC3 antagonist, MCE, New Jersey, USA), 50 μM ITSA-1 (an HDAC3 agonist, MCE, New Jersey, USA), 10 μM Stattic (a STAT3 antagonist, MCE, New Jersey, USA), and 25 nM rapamycin (an mTOR antagonist, MCE, New Jersey, USA) for 30 min before the addition of ConA (20 μg/ml, Sigma, St. Louis, MO, USA) and butyrate (0.5mM, Sigma, St. Louis, MO, USA). The suspensions were incubated at 37°C with 5% CO_2_ for 44 h, and the optical density value was later determined. Each assay used a repeat of 6 wells.

### Measurements of Antioxidant Activity and Lipid Peroxidation

Cecal portions (*n* = 6) were collected for antioxidant activity analysis. The superoxide dismutase (GSH-Px), catalase (CAT), glutathione peroxidase (GSH-Px), total antioxidant capability (T-AOC), and the malondialdehyde (MDA) analyses were measured by using commercial kits (Beyotime Co., Ltd., Shanghai, China). The experimental protocol was carried out according to the instructions. Each sample was tested three times.

### Enzyme-Linked Immunosorbent Assay

The cecal IL-10, IL-6, TNF-α, and IFN-γ levels were detected by ELISA (Uscn Life Science, Inc.). The experimental program was completed according to the instructions (intra-assay coefficient of variation = 7.9%). Each sample was tested in triplicate.

### PAS Staining and Immunohistochemical Staining

Six cecum sections (5 μm in thickness) were randomly selected for each sample, and at least 60 fields were photographed. In each field, the five longest villi were selected. The data were analyzed by measuring the number of goblet cells per 100 absorbed cells from periodic acid–Schiff (PAS) staining by using Image-Pro Plus software.

For immunohistochemical staining, primary antibodies (rabbit anti-PCNA,1:500, Abcam, Cambridge, UK; rabbit anti-MUC-2, 1:180, Abcam, Cambridge, UK; rabbit anti-sIgA, 1:200, Bethyl laboratories, Texas, USA; and rabbit anti-GPR43, 1:1,000, Abcam, Cambridge, UK) were incubated with the sections overnight at 4°C and visualized by incubating 0.05% 3,3-diaminobenzidine tetrahydrochloride (Sigma, St. Louis, MO, USA) and 0.003% hydrogen peroxide. Positive cells in five cross-sections were randomly selected for each sample, and at least 25 fields were counted. The integrated optical density (IOD) was measured by using Image-Pro Plus software.

### Western Blot Analysis

The proteins (*n* = 5) of cecum were extracted with RIPA lysis buffer, and the concentration with bicinchoninic acid kit (Beyotime, Wuhan, China) was determined. Then, equal amounts of protein in each group were added to SDS-polyacrylamide gel, transferred onto polyvinylidene fluoride membranes, and blocked for 1 h using 5% skimmed milk. Subsequently, primary antibodies including anti-claudin-1 (rabbit, 1:1,000, Invitrogen, California, USA), anti-occludin (rabbit, 1:1,000, Invitrogen, California, USA), anti-ZO-1 (rabbit, 1:1,000, Invitrogen, California, USA), anti-phospho-STAT3 (1:1,000, Abcam, Cambridge, UK), anti-cyclin D1 antibody (mouse, 1:200, Abbexa, Cambridge, UK), anti-HDAC3 antibody (rabbit, 1:1,000, Abcam, Cambridge, UK), anti-GPR43 antibody (rabbit, 1:1,000, Abcam, Cambridge, UK), or anti-β-actin (mouse, 1:4,000; Co Win Biotech Co., Inc, Beijing, China) were incubated with the membranes overnight at 4°C. Then, horseradish peroxidase-conjugated goat anti-mouse/rabbit antibody (1:8,000; Co Win Biotech Co., Inc. Beijing, China) was used to incubate the membranes for 2 h. The IOD of the target bands was measured by using ImageJ software (version 4.0.2; Scion Corp., Frederick, MD, USA) and normalized to the corresponding β-actin values. Each sample was tested in triplicate.

### Microbial Sequencing

The total DNA (*n* = 4) of cecum was extracted with QIAamp DNA Stool Mini Kit (Hilden, Germany). The V3–V4 region of the 16S rRNA gene was amplified by using PCR, and the detailed method was modified as previously described (18).

After data filtering, data rarefying was used, and all samples were rarefied to even out the sequencing depth based on the sample having the lowest sequencing depth (read counts = 26191). Hence, we conducted data rarefying to keep validity of all downstream analyses. According to UCLUST, the effective reads of each sample were clustered into operational taxa with 97% sequence similarity, and the QIIME method was used to identity the key bacteria at the phylum level or genus level. Then, β-diversity was estimated by calculating the weighted UniFrac distance, visualized by principal coordinate analysis (PCoA), and plotted by the “Vegan” and “GGplot2” software packages in R software (version 3.4.4). PERMANOVA (similarity analysis) was used to evaluate the significance of microbial structural differentiation between five groups. The R package was “pure”. The functional predictions based on 16S rRNA gene sequencing was estimated by Unobserved States 1 (PICRUSt 1) method (19) and visualized by statistical analysis of taxonomic and functional profiles software (20). Spearman’s correlations (*R* > 0.5, *p* < 0.05) and Mantel test was performed to examine the linkage between phenotypic variables and microbial communities (richness, Chao1 index, and Shannon index) or cecal butyrate concentration. The analysis method was performed according to a previously described method (21).

### Metabolomics Profiling

For metabolomics analysis, the LC-MS analyses were performed on a quadrupole-time-of-flight 6510 mass spectrometer (Agilent Technologies, Santa Clara, CA, USA) with an electrospray ionization source (22).

### Short-Chain Fatty Acid Extraction and Analysis

For VFA analysis, we collected fresh cecum content (*n* = 6) samples and stored them at –80°C. The preparation of supernatant was mentioned in previous studies (23). Then, the gas chromatograph (Agilent 6890N, Agilent Technologies, Inc., Beijing, China) was filled with a supernatant to determine the concentrations of acetate, propionate, and butyrate.

### Statistical Analysis

Data are presented as mean ± standard error of the mean (SEM) and analyzed using SPSS 25.0 software (SPSS, Chicago, IL, USA). Differences between groups were statistically analyzed using one-way ANOVA, and means were compared using Duncan’s multiple-range test. The results were considered statistically significant when the *P*-value was <0.05. Correlation analysis, expressed as Spearman’s coefficient, was performed to determine the correlations between the abundance of microbiota and intestinal development.

## Results

### Effect of Different Monochromatic Light Combinations on Altered Gut Microbiota Composition

#### Sequencing Overview

A total of 20 samples were obtained from five groups (*n* = 4) of chicks and subsequently sequenced to generate V3–V4 16S rRNA gene profiles. A total of 219,760, 242,947, 245,627, 273,814, and 271,351 raw reads were obtained for WW, RR, GG, BB, and G→B, respectively. There was an average of 32,012, 27,997, 34,220, 30,811, and 50599 clean reads in WW, RR, GG, BB, and G→B, respectively.

#### Bacterial Diversity and Community Structure

**Figure 2:**
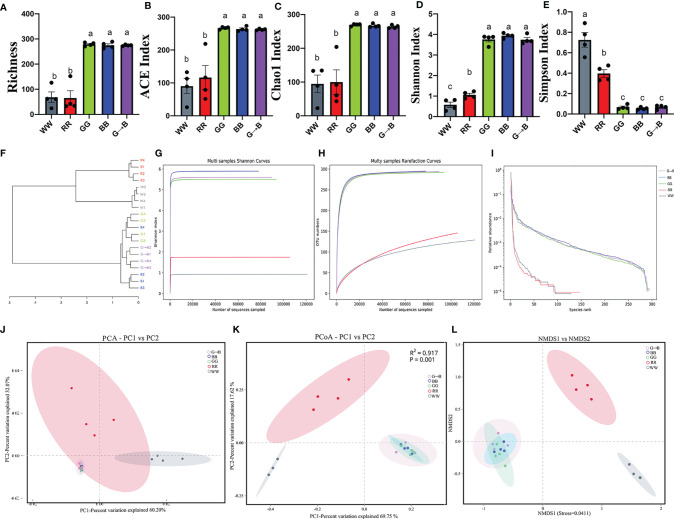
Richness **(A)**, ACE index **(B)**, Chao1 index **(C)**, Shannon index **(D)**, Simpson index **(E)**, dendrogram analysis **(F)**, Shannon curves **(G)**, rarefaction curves **(H)**, operational taxonomic unit (OTU) rank curves **(I)**, principal component analysis **(J)**, PCoA score plot **(K)**, and nonmetric multidimensional scaling score plot based on the Weighted UniFrac distance plot based on the OTU of the gut microbe **(L)** in the chick cecum of the WW, RR, GG, BB, and G→B groups at P42. WW, white light; RR, red light; GG, green light; BB, blue light; G→B, green light and blue light combination. The results are presented as means ± SEM. Different letters indicate significant differences between the treatments at the same age (*P* < 0.05).

As shown in **Figure 2A**, the richness of G→B was higher than WW and RR by 302.20–320.69% (*P* < 0.001) and lower than GG and BB by 0.09–1.55%, but there was no significant difference between GG, BB, and G→B (*P* > 0.05). In addition, as shown in **Figures 2B–E**, the quantitative perspective analysis suggested that the ACE, Chao1, and Shannon indexes were significantly increased by 125.65–190.82% (*P* < 0.001), 163.88–178.87% (*P* < 0.001), and 253.72–551.11% (*P* < 0.001) in G→B compared with WW and RR. However, the ACE, Chao1, and Shannon indexes were decreased by 0.50–5.29% in G→B compared with GG and BB (*P* > 0.05), while the Simpson index was markedly decreased by 82.89–90.64% (*P* < 0.001) in G→B compared with WW and RR and increased by 1.96–25.27% (*P* > 0.05) compared with GG and BB. A dendrogram analysis (**Figure 2F**) and PCoA plot (**Figure 2J**) based on weighted-unifrac distance showed distinct clustering between the WW, RR, GG, BB, and G→B (PREMANOVA: *R* ^2 =^ 0.917, *P* = 0.001); there was a close connection between the GG, BB, and G→B (**Figures 2K, L**). Furthermore, the microbiota in samples from the RR was grouped distinctly, and the distance between the RR and WW was less than that between the WW and GG, BB, or G→B. By comparing the shannon curves, rarefaction curve and OTU rank curves between the five groups, we found that the new OTUs and Shannon index declined with the increase in the sequencing number, which indicated that our samples covered most microbial species information (**Figures 2G–I**).

### Abundance and Significant Difference Between Five Groups at the Phylum Level

**Figure 3:**
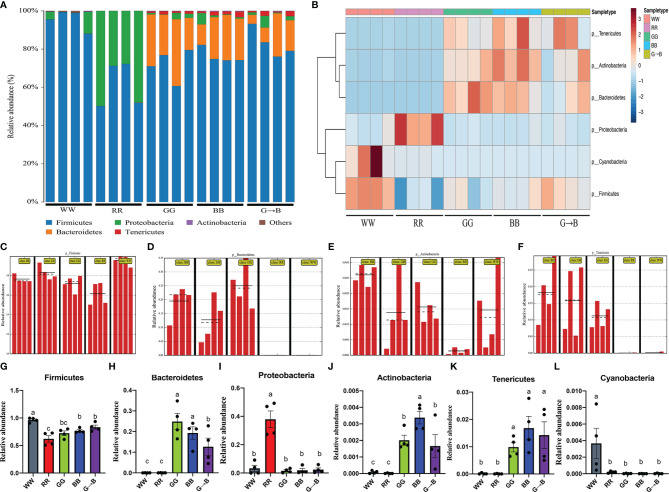
Relative contribution of the top 10 phyla in chick cecum microbe of WW, RR, GG, BB, and G→B groups at P42 **(A)**. Heat map of the redundancy analysis‐identified key phyla that were significantly altered in the chick cecum microbe of WW, RR, GG, BB, and G→B groups at P42 **(B)**. Linear discriminant analysis effect size approach identifying the different phyla in the chick cecum microbe of WW, RR, GG, BB, and G→B groups at P42 **(C–F)**. Taxonomic profiles of the notably significantly different bacteria at the phylum level in the chick cecum microbe of WW, RR, GG, BB, and G→B groups at P42 **(G–L)**. The results are presented as means ± SEM. Different letters indicate significant differences between the treatments at the same age (*P* < 0.05).

At the phylum level, the dominant bacteria harbored in chick cecum were *Firmicutes* (WW: 95.48%, RR: 51.99%, GG: 72.03%, BB: 76.37%, G→B: 82.99%), *Bacteroidetes* (WW: 0.04%, RR: 0.12%, GG: 25.01%, BB: 19.51%, G→B: 12.77%), and *Proteobacteria* (WW: 3.91%, RR: 47.85%, GG: 1.62%, BB: 2.00%, G→B: 2.50%). *Actinobacteria*, *Tenericutes*, and *Cyanobacteria* made up a smaller percentage in chick cecum (**Figure 3A**). To identify the specific bacterial phyla associated with WW, RR, GG, BB, and G→B, we conducted the heat map analysis (**Figure 3B**) and linear discriminant analysis effect size (LEfSe) analysis (**Figures 3C–F**). The LEfSe analysis showed that 4 taxa biomarkers in the five groups were identified with linear discriminant analysis (LDA) score >4 and *P* < 0.05. Subsequently, we summarized the relative abundance of the phylum in detail. As shown in **Figures 3D, H**, the relative abundance of *Bacteroidetes* was more abundant in GG than in G→B (*P* = 0.010). As shown in **Figures 3E, J**, the relative abundance of *Actinobacteria* was more abundant in BB than in G→B (*P* = 0.006). However, the relative abundance of *Firmicutes* (**Figures 3C, G**), *Proteobacteria* (**Figure 3I**), *Tenericutes* (**Figures 3F, K**), and *Cyanobacteria* (**Figure 3L**) had no significant difference between GG, BB, and G→B (*P* > 0.005). The heat map revealed the same pattern in these phyla (**Figure 3B**).

### Abundance and Significant Difference Between Five Groups at the Genus Level

**Figure 4:**
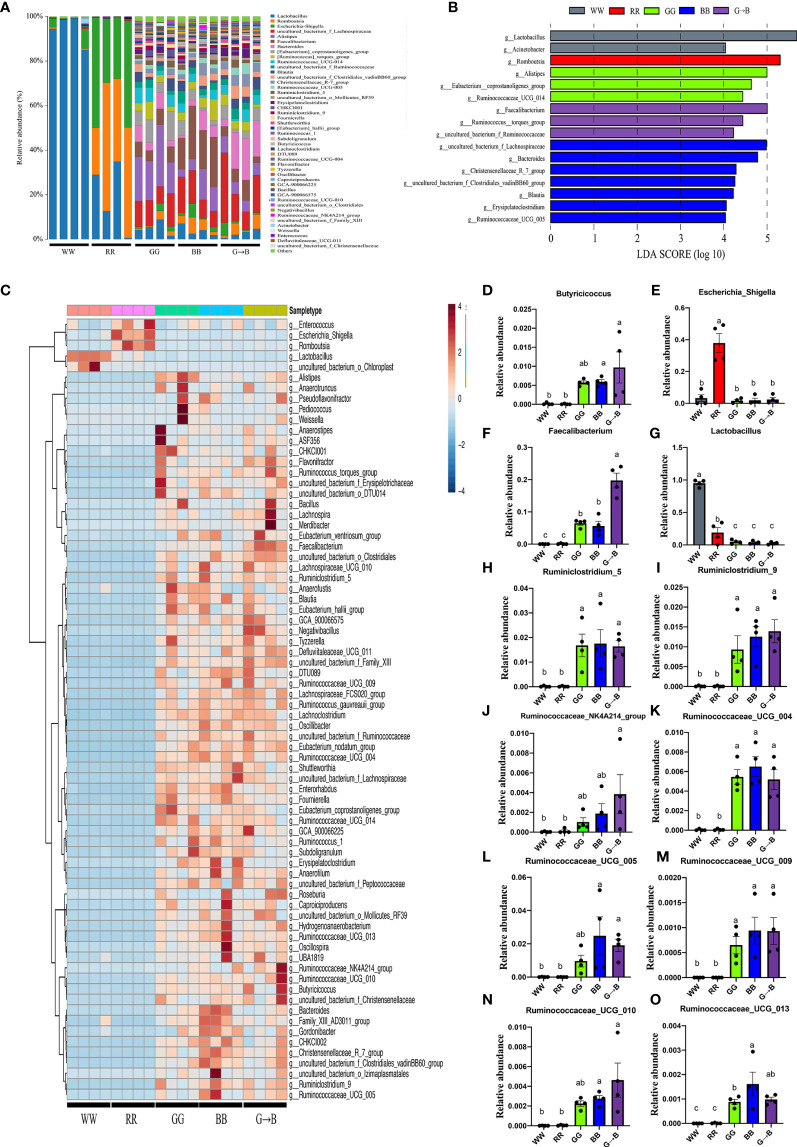
Forty-five genera that were present at a relative abundance of >0.1% in the cecum microbe of the WW, RR, GG, BB, and G→B groups were identified **(A)**, linear discriminant analysis effect size approach identifying the different genera in the chick cecum microbe of the WW, RR, GG, BB, and G→B groups at P42 **(B)**. Heat map of the redundancy analysis identifying the most differentially abundant genus in cecum microbiota in response to different monochromatic light combinations **(C)**. Taxonomic profiles of the notably significantly different bacteria at the genus level in the chick cecum of WW, RR, GG, BB, and G→B groups at P42 **(D–O)**. WW, white light; RR, red light; GG, green light; BB, blue light; G→B, green light and blue light combination. The results are presented as means ± SEM. Different letters indicate significant differences between the treatments at the same age (*P* < 0.05).

In total, 45 generas were identified, which were present at a relative abundance of >0.1%, and *Lactobacillus*, *Escherichia-Shigella*, and *Romboutsia* accounted for >98% of the sequences (**Figure 4A**). Next, LEfSe analysis was used to select the significantly different genera. It was found that 16 taxa biomarkers in five groups were identified with LDA score >4 and *P* < 0.05, which mainly belong to the phyla of *Firmicutes* and *Bacteroidetes* (**Figure 4B**). The redundancy analysis (**Figure 4C**) further identifies the difference of the gut microbiota that responded to different wavelength lights. Subsequently, we summarized the relative abundance of the genera in detail. The relative abundance of *Lactobacillus* in WW was the highest among all groups (**Figure 4G**). As shown in **Figure 4F**, the relative abundance of *Faecalibacterium* was significantly higher in G→B than in WW, RR, GG, and BB (*P* < 0.001). In addition, the relative abundance of *Butyricicoccus* (**Figure 4D**, *P* = 0.002–0.003), *Ruminiclostridium_5* (**Figure 4H**, *P* = 0.004), *Ruminiclostridium_9* (**Figure 4I**, *P* < 0.001), *Ruminococcaceae_NK4A214_group* (**Figure 4J**, *P* = 0.017–0.020), *Ruminococcaceae_UCG_004* (**Figure 4K**, *P* < 0.001), *Ruminococcaceae_UCG_005* (**Figure 4L**, *P* = 0.030), *Ruminococcaceae_UCG_009* (**Figure 4M**, *P* = 0.003), *Ruminococcaceae_UCG_010* (**Figure 4N**, *P* < 0.001), and *Ruminococcaceae_UCG_013* (**Figure 4O**, *P* = 0.008–0.009) was significantly higher in G→B than in WW and RR, while there was no significant difference between GG, BB, and G→B (*P* > 0.05). Besides this, as shown in **Figure 4E**, the relative abundance of *Escherichia_Shigella* in RR was significantly higher than WW, GG, BB, and G→B, respectively (*P* < 0.001).

### Functional Prediction Analysis in Five Groups

**Figure 5:**
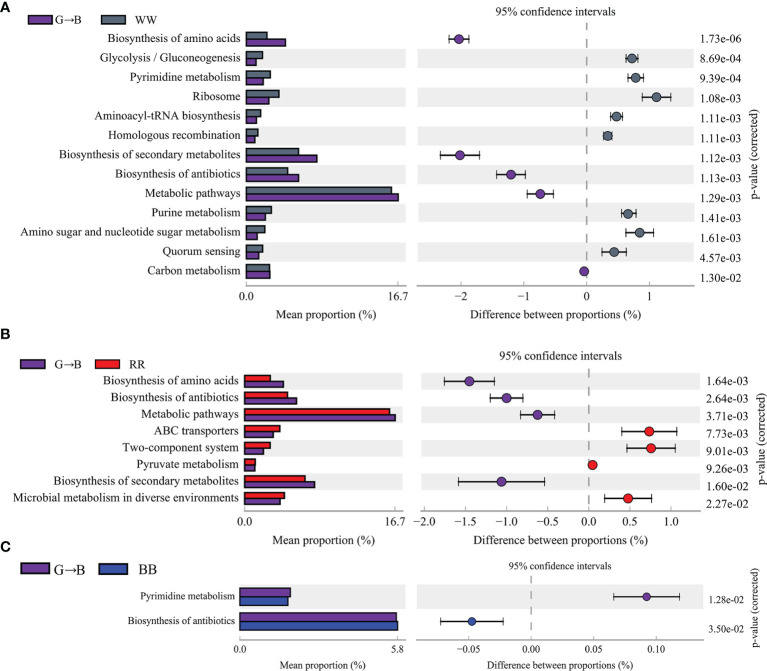
Comparison of Kyoto Encyclopedia of Genes and Genomes pathway enrichment between the groups by statistical analysis of taxonomic and functional profiles at level 3. WW *vs*. G→B **(A)**, RR *vs*. G→B **(B)**, and BB *vs*. G→B **(C)**. WW, white light; RR, red light; GG, green light; BB, blue light; G→B, green light and blue light combination. The results are presented as means ± SEM. Different letters indicate significant differences between the treatments at the same age (*P* < 0.05).

The presumptive functions of cecum microbiota were illustrated using PICRUSt (**Figures 5A–C**). In comparison with the WW and G→B, the “biosynthesis of amino acids”, “biosynthesis of secondary metabolites”, “biosynthesis of antibiotics”, and “metabolic pathways” predicted that the function of the Kyoto Encyclopedia of Genes and Genomes (KEGG) level 3 pathway was increased in the G→B. The comparison with the RR and G→B showed similar results. Compared with BB, “pyrimidine metabolism” predicted that the function of the KEGG level 3 pathway in G→B was increased and that “biosynthesis of antibiotics” in G→B was decreased. However, there was no significant difference in the KEGG level 3 pathway between GG and G→B.

### Effect of Different Monochromatic Light Combinations on Altered Gut Microbiota Metabolite Composition

**Figure 6:**
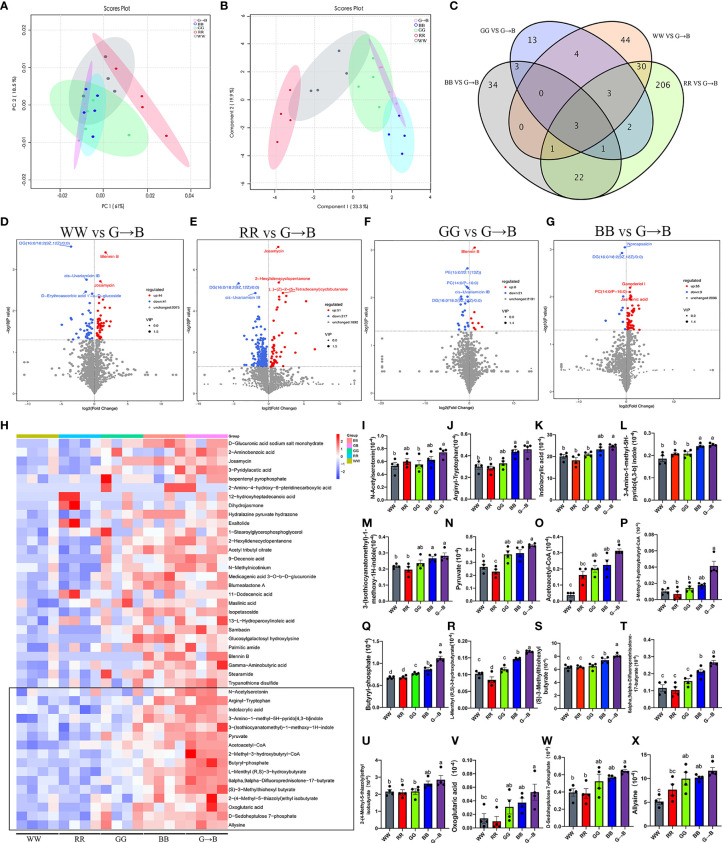
The β-diversity of the principal component analysis **(A)** and partial least squares discriminant analysis based on the microbiota metabolites **(B)** in the WW, RR, GG, BB, and G→B groups at P42. Venn diagram based on the microbiota metabolites **(C)** in the WW, RR, GG, BB, and G→B groups. Volcano plot based on the differential metabolite screening compared with the WW and G→B groups **(D)**. Volcano plot based on the differential metabolite screening compared with the RR and G→B groups **(E)**. Volcano plot based on the differential metabolite screening compared with the GG and G→B groups **(F)**. Volcano plot based on the differential metabolite screening compared with the BB and G→B groups **(G)**. Heat map of the redundancy analysis‐identified 49 key metabolites that were significantly altered in the chick cecum microbe of G→B group at P42 **(H)**. The relative abundance of N-acetyserotomimin **(I)**, arginyl-tryptophan **(J)**, indolacrylic acid **(K)**, 3-amino-1-methyl-5H-pyrido[4,3-b]indole **(L)**, 3-(isothiocyanatomethyl)-1-methoxy-1H-indole **(M)**, pyruvate **(N)**, acetoacetyl-CoA **(O)**, 2-methyl-3-hydroxybutyryl-CoA **(P)**, butyryl-phosphate **(Q)**, L-menthyl (R,S)-3-hydroxybutyrate **(R)**, (S)-3-methylthiohexyl butyrate **(S)**, 6alpha,9alpha-difluoroprednisolone-17-butyrate **(T)**, 2-(4-methyl-5-thiazolyl)ethyl isobutyrate **(U)**, oxoglutaric acid **(V)**, D-sedoheptulose 7-phosphate **(W)**, and allysine **(X)** in the WW, RR, GG, BB, and G→B groups at P42 in the cecum microbiota based on the heat map results. WW, white light; RR, red light; GG, green light; BB, blue light; G→B, green light and blue light combination. The results are presented as means ± SEM. Different letters indicate significant differences between the treatments at the same age (*P* < 0.05).

As shown in **Figures 6A, B**, the score plots from the PCA and PLS-DA showed an obvious separation between WW, RR, GG, BB, and G→B. The Venn diagram and volcano plot indicated that different light treatments resulted in different metabolite changes (**Figures 6C–G**). Compared with WW, there were 85 metabolites changed in G→B, in which 44 metabolites were upregulated and 41 metabolites were downregulated. Compared with RR, there were 268 metabolites changed in G→B, in which 51 metabolites were upregulated and 217 metabolites were downregulated. Compared with GG, there were 29 metabolites changed in the G→B, in which 8 metabolites were upregulated and 21 metabolites were downregulated. Compared with GG, there were 64 metabolites changed in G→B, in which 55 metabolites were upregulated and 9 metabolites were downregulated. Next, 44 metabolites with the significant difference in the 5 light treatment groups were analyzed (**Figure 6H**). There was a significant increase in the contents of N-acetyserotomimin (**Figure 6I**, *P* = 0.014–0.018), arginyl-tryptophan (**Figure 6J**, *P* = 0.003–0.010), indolacrylic acid (**Figure 6K**, *P* = 0.002–0.042), 3-amino-1-methyl-5H-pyrido[4,3-b]indole (**Figure 6L**, *P* = 0.002–0.020), 3-(isothiocyanatomethyl)-1-methoxy-1H-indole (**Figure 6M**, *P* = 0.005–0.023), pyruvate (**Figure 6N**, *P* = 0.000–0.004), acetoacetyl-CoA (**Figure 6O**, *P* = 0.000–0.020), 2-methyl-3-hydroxybutyryl-CoA (**Figure 6P**, *P* = 0.003–0.041), butyryl-phosphate (**Figure 6Q**, *P* = 0.000), L-menthyl (R,S)-3-hydroxybutyrate (**Figure 6R**, *P* = 0.000–0.009), (S)-3-methylthiohexyl butyrate (**Figure 6S**, *P* = 0.000–0.025), 6alpha,9alpha-difluoroprednisolone-17-butyrate (**Figure 6T**, *P* = 0.000–0.030), 2-(4-methyl-5-thiazolyl) ethyl isobutyrate (**Figure 6U**, *P* = 0.007–0.012), oxoglutaric acid (**Figure 6V**, *P* = 0.006–0.027), D-sedoheptulose 7-phosphate (**Figure 6W**, *P* = 0.003–0.007), and allysine (**Figure 6X**, *P* = 0.001–0.023) in G→B compared to WW, RR, and GG.

### Effect of Different Monochromatic Light Combinations on Altered Metabolic Pathway

To identify how the changes in intestinal microbiota metabolites affect host signaling pathways, we classified the annotation results of differential metabolites according to the pathway types in KEGG. As shown in **Supplementary Figures S1A, B**, G→B mainly affects the following signaling pathways: sphingolipid signaling pathway, EGFR tyrosine kinase inhibitor resistance, MAPK signaling pathway, ErbB signaling pathway, calcium signaling pathway, chemokine signaling pathway, NF-kappa B signaling pathway, VEGF signaling pathway, and apelin signaling pathway. Meanwhile, the G→B-induced pathway changes were closely linked to the Natural cell-mediated cytotoxicity, Th1 and Th2 cell differentiation, Th17 cell differentiation, and T cell receptor signaling pathway. Therefore, we suggested that changes in the microbial composition of G→B can lead to changes in the metabolic pathways and ultimately affect the physiological activities of the chicken.

### Effect of Different Monochromatic Light Combinations on the Antioxidant Capacity of the Cecum

**Figure 7:**
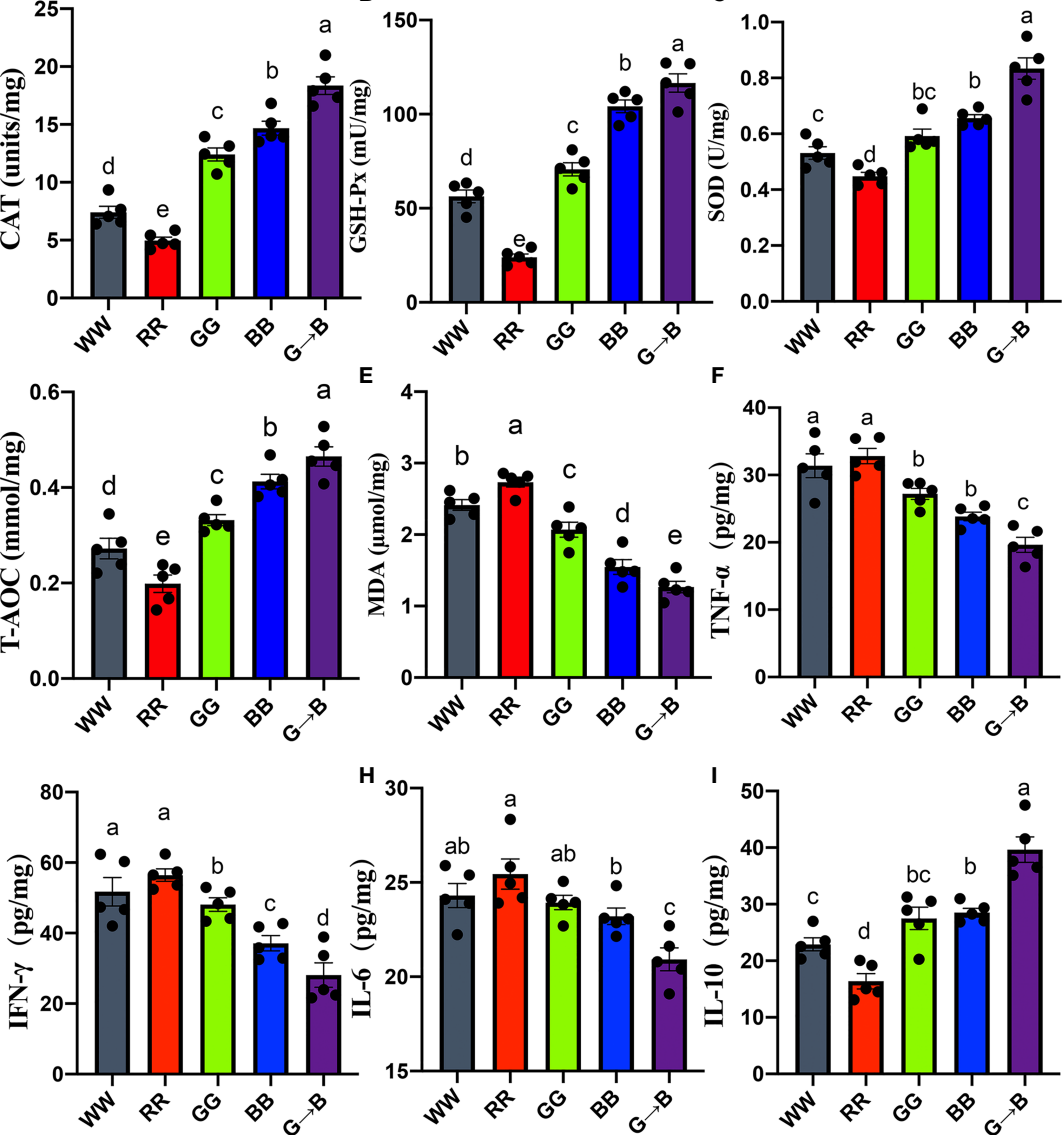
Effect of different monochromatic light combinations on CAT **(A)**, GSH-Px **(B)**, SOD **(C)**, T-AOC **(D)**, MDA **(E)**, pro-inflammatory cytokine TNF-α **(F)**, IFN-γ **(G)**, IL-6 **(H)**, and anti-inflammatory cytokine IL-10 **(I)** concentrations in the cecum at P42. WW, white light; RR, red light; GG, green light; BB, blue light; G→B, green light and blue light combination. The results are presented as means ± SEM. Different letters indicate significant differences between the treatments at the same age (*P* < 0.05).

As shown in **Figures 7A–D**, G→B significantly improved the antioxidant enzymes and T-AOC compared with WW, RR GG and BB 19.93–70.86% (CAT, *P* < 0.001), 41.01–125.78% (GSH-Px, *P* < 0.001), 27.77–76.31% (SOD, *P* < 0.001), and 31.92–171.36% (T-AOC, *P* = 0.001) in the cecum. However, the MDA content, which was a lipid peroxidation production, was significantly decreased in G→B and significantly increased in RR (**Figure 7E**).

### Effects of Different Monochromatic Light Combinations on Cecal Cytokine Levels

As shown in **Figures 7F–H**, G→B caused a decrease in cecum pro-inflammatory cytokine TNF-α (21.45–67.14%, *P* = 0.000–0.019), IFN-γ (32.06–100.85%, *P* = 0.000–0.035) and IL–6 (10.93–21.64%, *P* = 0.000–0.013) level. As shown in **Figure 7I**, G→B caused an increase in anti-inflammatory cytokine IL–10 (39.11–142.04%, *P* = 0.000) levels compared with WW, RR, GG, and BB, respectively. In contrast, the proinflammatory cytokine IL–6, TNF-α, and IFN-γ levels in RR were the highest among the other four groups.

### Effects of Different Monochromatic Light Combinations on the Cecal Mucosal Immune Function

**Figure 8:**
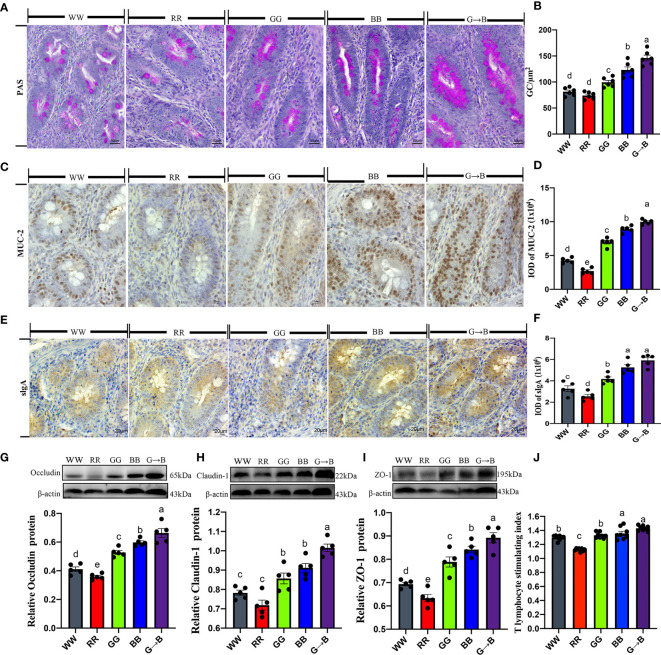
Effects of different monochromatic light combinations on periodic acid-Schiff staining of cecum tissue sections **(A)**, the number of goblet cells per square micrometer in the cecum (scale: 50 μm) **(B)**, immunohistochemical staining of MUC-2 in cecum sections (scale: 20 μm) **(C)**, the integrated optical density (IOD) of MUC-2‐positive cells **(D)**, immunohistochemical staining of sIgA^+^ cells in cecum sections (scale: 20 μm) **(E)**, the IOD of sIgA^+^ cells **(F)**, occludin, claudin‐1, and ZO‐1 protein expression **(G–I)**, and cecal tonsil T proliferation stimulation index **(J)** in chicks at P42. WW, white light; RR, red light; GG, green light; BB, blue light; G→B, green light and blue light combination. The results are presented as means ± SEM. Different letters indicate significant differences between the treatments at the same age (*P* < 0.05).

To investigate whether different monochromatic light combinations could affect the mucosal immune function of the cecum, we detected the changes in goblet cell numbers, expression of mucin, and tight junction proteins in the mucosal epithelium. As shown in **Figures 8A, B**, the red goblet cells showed by PAS staining were mainly distributed in the villi of the cecum. The number of goblet cells per 100 absorbed cells in G→B was increased by 18.54–97.30% (*P* = 0.000–0.001) compared with WW, RR, GG, and BB. Similar results were observed in the IOD of MUC-2 which was significantly increased by 12.02-267.75% (*P* = 0.000–0.002) in G→B compared with WW, RR, GG, and BB (**Figures 8C, D**). In addition, the IOD of sIgA+ cells in G→B was significantly increased by 12.46–132.66% (*P* = 0.000) than WW, RR, and GG (**Figures 8E, F**). Western blot analysis revealed that the occludin protein was significantly upregulated by 11.05–84.80% (*P* = 0.000–0.015) in G→B compared with WW, RR, GG, and BB (**Figure 8G**). Similar results were observed in the expression level of claudin-1 and ZO-1 protein (**Figures 8H, I**). *In vitro*, we isolated T lymphocyte *in vitro* from chicks cecal tonsil to assess whether different monochromatic light combinations could affect intestinal lymphocyte proliferation. As shown in **Figure 8J**, the stimulating index of ConA-activated T lymphocyte in G→B was 7.54% to 27.11% higher than that obtained with the other light treatment groups (*P*= 0.000–0.002). Additionally, there was a strong correlation between the butyrate concentration in the cecum content and the stimulation index of T lymphocyte (r = 0.8959, *P* = 0.040).

### Relationships Between Phenotypic Variables, Bacterial Communities, and Cecal Butyrate Concentration

**Figure 9:**
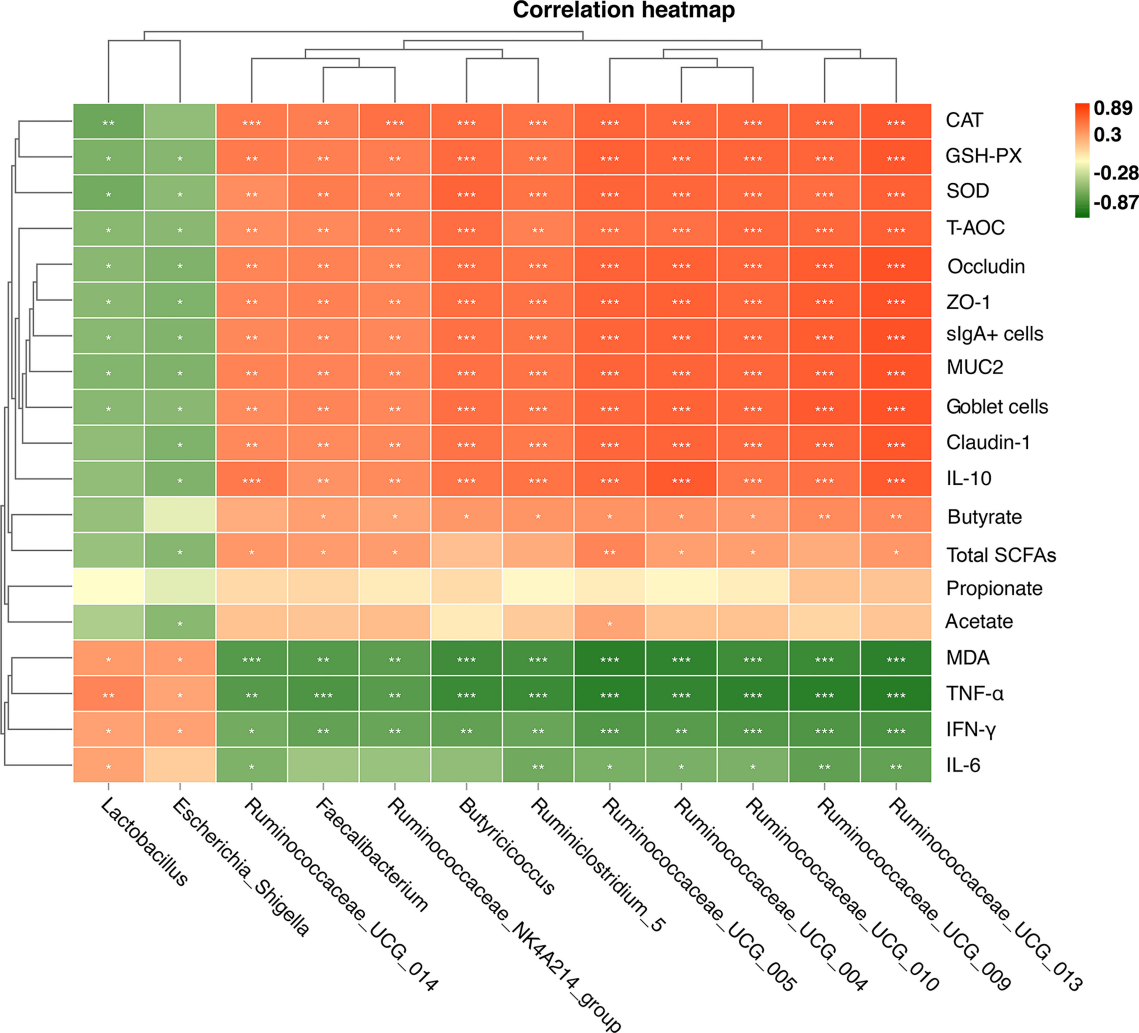
Heat map of Spearman’s correlation between the relative abundances of 12 key genus and phenotypic variables **(A)**. These 12 key genera were notably significantly different bacteria at the genus level in the chick cecum of WW, RR, GG, BB, and G→B groups at P42. The colors range from green (negative correlations) to red (positive correlations). WW, white light; RR, red light; GG, green light; BB, blue light; G→B, green light and blue light combination. *P < 0.05; **P < 0.01; ***P < 0.001.

The shifts in microbial community were tightly linked to phenotypic variables as revealed by the Mantel test. As shown in **Figure 9**, Spearman’s correlation analysis showed that cecal butyrate concentration was positively (*P* < 0.05 or *P* < 0.01) correlated with cecal *Ruminiclostridium_5*, *Butyricicoccus*, *Faecalibacterium*, *Ruminococcaceae_NK4A214_group*, *Ruminococcaceae_UCG_010*, *Ruminococcaceae_UCG_004*, *Ruminococcaceae_UCG_005*, *Ruminococcaceae_UCG_013*, and *Ruminococcaceae_UCG_009.* The cecal tight junction protein (ZO-1, occludin, and claudin-1) was positively (*P* < 0.05 or *P* < 0.01) correlated with cecal *Ruminiclostridium_5*, *Butyricicoccus*, *Faecalibacterium*, *Ruminococcaceae_NK4A214_group*, *Ruminococcaceae_UCG_010*, *Ruminococcaceae_*UCG_004, Ruminococcaceae_UCG_005, Ruminococcaceae_UCG_014, Ruminococcaceae_UCG_015, *and* Ruminococcaceae_UCG_009 *but negatively correlated with* Lactobacillus *and* Escherichia-Shigella. *The anti-inflammation cytokine IL-10 was positively* (P < 0.05 or P < 0.01) *correlated with* cecal Ruminiclostridium_5, Butyricicoccus, Faecalibacterium, Ruminococcaceae_NK4A214_group, Ruminococcaceae_UCG_010, Ruminococcaceae_UCG_004, Ruminococcaceae_UCG_005, Ruminococcaceae_UCG_014, Ruminococcaceae_UCG_015, *and* Ruminococcaceae_UCG_009 *but negatively correlated with* Escherichia-Shigella. *The results for antioxidant enzymes were similar.*

As shown in **Supplementary Figure S2**, Mantel correlation analysis showed that the cecum microbiome composition or cecal butyrate concentration was significantly positively correlated (*r* > 0.5, *P* < 0.05) with tight junction protein (ZO-1, claudin-1, and occludin), antioxidant enzymes (CAT, GSH-Px, SOD, and T-AOC), anti-inflammation (IL-10), and sIgA, while it was negatively correlated (*r* < -0.5, *P* < 0.05) with MDA and pro-inflammation (TNF-α, IFN-γ, and IL-6).

### The GPR43/HDAC3/STAT/mTOR/Signaling Pathways Are Involved in Butyrate-Mediated G→B-Induced Cecal Tonsil T Lymphocyte Proliferation

**Figure 10:**
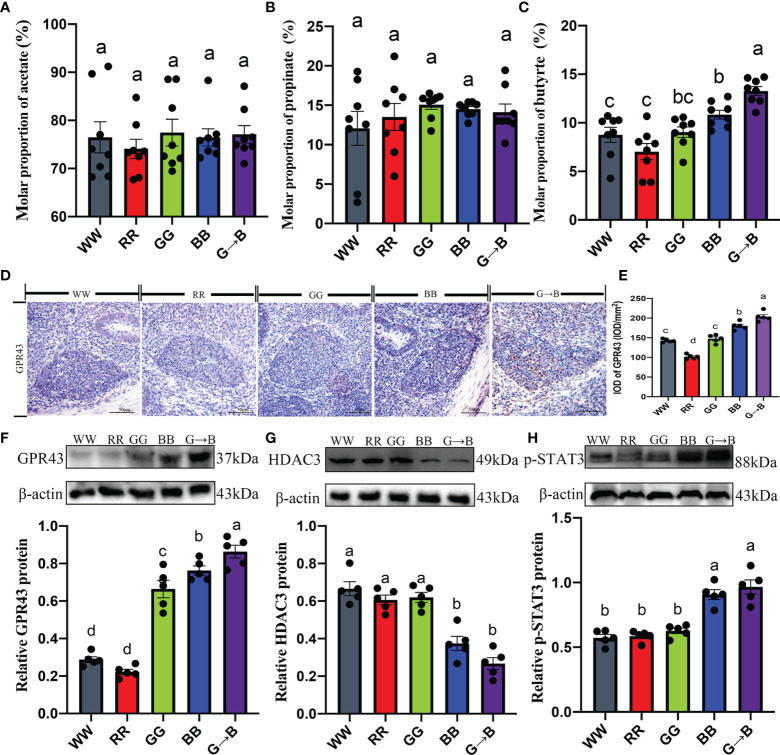
Effects of different monochromatic light combinations on the cecum acetate concentration **(A)**, cecum propionate concentrations **(B)**, cecum butyrate concentration **(C)**, photomicrographs of the immunostaining of butyrate receptor subtypes GPR43 in the cecal tonsil at P42 **(D)**, the integrated optical density of GPR43-positive cells **(E)**, GPR43 protein expression **(F)**, HDAC3 protein expression **(G)**, and p-STAT3 protein expression **(H)** in the cecal tonsil at P42. WW, white light; RR, red light; GG, green light; BB, blue light; G→B, green light and blue light combination. The results are presented as means ± SEM. Different letters indicate significant differences between the treatments at the same age (*P* < 0.05).

As shown in **Figure 10C**, the chicks that were exposed to G→B showed a significantly higher content of butyrate than WW, RR, and GG by 22.34–88.80% (*P* = 0.000–0.010). However, we found that the contents of acetate and propionate in WW, RR, GG, BB, and G→B had no significant difference (**Figures 10A, B**).

To investigate whether butyrate could mediate G→B-induced cecal tonsil T lymphocyte proliferation, the signaling pathway was studied using both *in vitro* and *in vivo* experiments. Compared with the WW, RR, GG, and BB, there was an obvious upregulation in the expression of GPR43 (13.52–200.42%, *P* = 0.000–0.026; **Figures 10D–F**), p-STAT3 (6.55–68.75%, *P* < 0.001; **Figure 10H**) as well as a significant downregulation in the expression of HDAC3 (40.26–149.30%, *P* = 0.000–0.028; **Figure 10G**) in the cecal tonsil of G→B. A ConA-induced cecal tonsil T lymphocyte model with or without butyrate administration was established *in vitro*.

**Figure 11:**
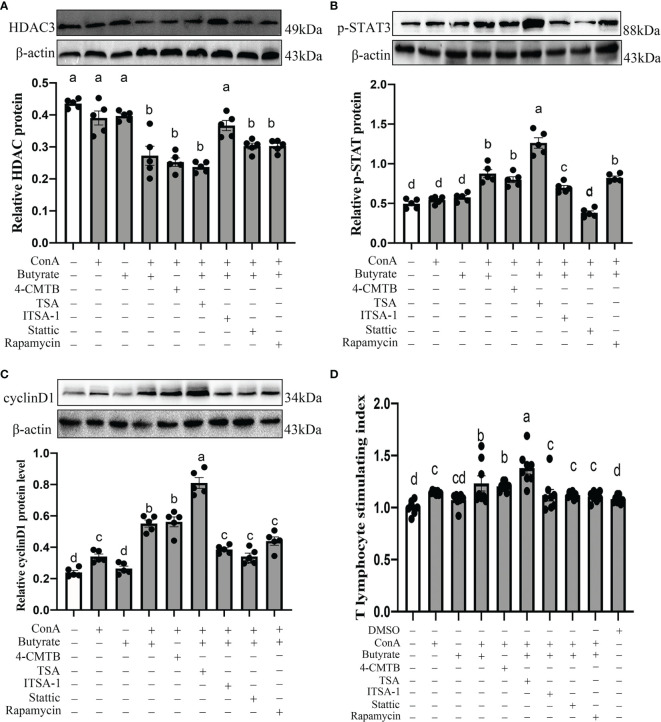
Effects of GPR43 agonist, HDAC3 antagonist, HDAC3 agonist, STAT antagonist, and mTOR antagonist on HDAC3 protein level **(A)**, p-STAT3 protein level **(B)**, cyclin D1 protein level **(C)**, and cecal tonsil T proliferation stimulation index **(D)**. 4-CMTB is a GPR43 agonist, TSA is a HDAC3 antagonists, ITSA-1 is a HDAC3 agonist, Stattic is a STAT3 antagonists, and rampamycin is a mTOR antagonists. Values with no common letters are significantly different (*P* < 0.05) from each other. p-STAT3, phosphorylated STAT3.

As shown in **Figure 11A**, we found that the pretreatment of T lymphocyte with 0.5 mM butyrate in response to ConA significantly induced an upregulation of p-STAT3 (76.53%, *P* < 0.001; **Figure 11B**) and cyclin D1 (130.07%, *P* < 0.001; **Figure 11C**) protein expression, improved the T lymphocyte stimulating index (23.25%, *P* < 0.001; **Figure 11D**), and decreased the HDAC3 (37.45%, *P* < 0.001; **Figure 11A**) protein level compared with the control group. Similarly, as shown in **Figures 11B–D**, pretreatment of T lymphocyte with ConA + 4-CMTB (GPR43 agonist) effectively mimicked the improving effect of butyrate and increased the T lymphocyte stimulating index (20.38%, *P* < 0.001), p-STAT3 (60.99%, *P* < 0.001), cyclin D1 (134. 56%, *P* < 0.001), and protein level and downregulated HDAC3 (42.05%, *P* < 0.001) protein expression compared with the control groups. Consistent with these, the promoting effect of butyrate on cyclin D1 protein expression and T lymphocyte proliferation was significantly strengthened by the HDAC3 antagonist TSA and blocked by HDAC3 agonist ITSA-1. Similarly, the administration of Stattic (a STAT3 antagonist) or rapamycin (a mTOR antagonist) countered the improving effect of butyrate and led to a downregulation of cyclin D1 (20.26–38.03%, *P* = 0.001) and T lymphocyte stimulating index (9.04-10.06, *P* =0.017–0.031) compared with the ConA + butyrate-treated groups. However, pre-treated T lymphocyte with rapamycin had no significant effect on p-STAT3 protein expression compared with ConA + butyrate-treated groups. Hence, butyrate exerted its pro-proliferation effects *via* the GPR43/HDAC3/STAT3/mTOR/cyclin D1 signaling pathway.

## Discussion

Through integrating the cecum 16sRNA gene amplicon sequencing and metabolomes, we investigated the cecal microbial composition under different monochromatic light combinations in chickens. In the present study, we found that *Firmicutes*, *Proteobacteria*, and *Bacteroidetes* were the most predominant active bacterial phyla regardless of the effects of the different wavelengths of light in the cecum. These results were consistent with the bacterial phylum profiles in chicks under 16S rRNA and metagenomic sequencing analysis (24). Although the same phyla were identified as dominant bacteria in 16S rRNA studies, their community structure is quite different between WW, RR, GG, BB, and G→B, which indicated that the interactions of the microbe had been changed under the regulation of different wavelengths and further shaped the different structures of the microbial community. It was also evidenced by the results of the PCoA and dendrogram analysis (**Figures 2F, K**). The difference in biological community composition has previously been observed to be associated with photoperiod (6) and light intensity (25). In addition, the diversity and richness of the cecal microbial population in G→B was significantly increased. A series of reports have clarified that the pathogenesis of irritable bowel syndrome is related to the loss of microbial diversity and richness. Thus, we suggested that G→B may be beneficial to the development of intestinal microbes and reduce the occurrence of intestinal diseases (26). However, what was the reason for the significant increase in cecal microbial diversity and richness in G→B? Our previous studies found that light wavelength could influence the rhythms of melatonin synthesis and clock gene expression in the central oscillator of chicks (27). In the previous study, we found that a green-and-blue monochromatic light combination could increase the plasma melatonin concentration in chickens (17). Melatonin, as a key endogenous factor in limiting free radical damage, was found to efficiently improve the reductive potential of tissues and fluids. In the present study, we found that G→B significantly increased the activities of GSH-Px, SOD, CAT, and T-AOC and reduced the MDA content or pro-inflammatory cytokine levels in the cecum of chickens compared with other monochromatic lights. In addition, some strong positive correlations between bacterial communities (richness, Shannon, and Chao1) and antioxidant enzyme (CAT, SOD, and GSH-PX), T-AOC, IL-10 were found in our study. Thus, we speculated that melatonin may provide protection against free radical damage and decrease the oxidative stress level. Oxidative stress level is an important factor regulating the composition and function of the intestinal microbiota in vertebrates (28). These findings suggested that a green-and-blue monochromatic light combination can adjust the circadian rhythm of chickens and then promote the pineal gland to secrete melatonin. Melatonin may transmit external light signals to intracellular molecules to improve the antioxidant capacity of chicken cecum, leading to a significant increase in the diversity and richness of cecal microorganisms in G→B. To confirm our hypothesis, pinealectomy should be carried out further to inhibit the secretion of melatonin and verify the core role of melatonin in G→B-induced changes in cecal microbiota composition.

Next, to better understand the effect of the different monochromatic light combinations on the characteristic changes of microbial colonization in the cecum, several genera as biomarkers were identified in the five light treatment groups. In the WW group, we observed that the abundance of *Lactobacillus* was the highest. In a previous study, it has been provided that *Lactobacillus* could efficiently ferment carbohydrate and inhibit the colonization of other bacteria by lowering the pH levels in the digestive tract (29). This may be the reason why the diversity of cecal microorganisms in the WW was lower than that of other light treatment groups. In the G→B, *Butyricicoccus* (30), *Ruminiclostridium* (31), and *Ruminococcaceae* (32), as SCFAs-producing bacteria, were enriched, which could be responsible for the elevated cecal SCFAs in the chick. In particular, *Butyricicoccus* could produce a significant amount of butyrate which has important immunomodulatory functions and act as modulators of chemotaxis and adhesion of immune cells (33). Additionally, a greater proportion of the genus *Faecalibacterium*, which has been characterized as an anti-inflammatory mediator (34), was observed in the G→B compared with WW, RR, GG, and BB. However, the pathogenic bacteria, such as *Escherichic_Shigella*, are prominent members in RR. Recent studies found that *Escherichic_Shigella* has been recognized to be negatively correlated with growth and fat digestibility (35) and became a significant cause of morbidity and mortality in the broiler (36).

Previous studies have shown that the core microbial community in the intestine is not determined by the specific intestinal microbial species but by the collective “enrichment function” contained in the community. Interestingly, we found that a higher abundance of genes involved in metabolism (like biosynthesis of amino acids and biosynthesis of secondary metabolites) were enriched in G→B as compared to WW and RR, indicating that more protein products might be generated by the G→B microbiome due to the higher ability to biosynthesize amino acids. In addition, the KEGG functions in the biosynthesis of antibiotics were enriched in the cecum of G→B, providing further evidence that chicks in G→B had a better immunity function. Our study also found that the number of sIgA+ cells, the MUC-2, and tight protein expression were all increased in G→B, indicating that the G→B chicks had a better intestinal immunity function. Furthermore, previous studies in our laboratory found that G→B can increase the level of NDV antibodies (10) in plasma and promote the B lymphocyte proliferation of broilers (17b), which supports our suggestion. Additionally, the intestinal tight protein (including ZO-1, occludin, and claudin-1), goblet cell numbers, MUC-2 protein, and sIgA+ cells were positively correlated with SCFA-producing bacteria (*Ruminococcacese*, *Butyricicoccus*, and *Faecalibacterium*). These results indicated that the colonization of SCFA-producing bacteria might be the principal factors affecting intestinal mucosal immune function.

Using metabolomics, we found that G→B significantly improved the pyruvate, acetoacetyl-CoA, 2-methyl-3-hydroxybutyryl-CoA, and butyryl-phosphate levels compared with the other four groups. Kayama et al. (16) found that *Faecalibacterium* could produce butyrate by carbohydrate fermentation *via* the conversion of pyruvate and acetyl-CoA. Acetyl-CoA is then converted to acetoacetyl-CoA and hydroxybutyryl-CoA, which eventually produces butyryl-phosphate. Butyryl phosphate is then converted to butyrate by the butyrate kinase enzyme. Our microbial sequencing results, which showed that the abundance of *Faecalibacterium* improved in G→B, supported this conjecture. Another important metabolite improved in the G→B group were lysine and its synthetic precursors, including oxoglutaric acid and D-sedoheptulose 7-phosphate. Previous studies have found that *Butyricimonas* and *Pseudoflavinofractor* could produce butyrate by lysine fermentation (2). These results implied that G→B promotes carbohydrate fermentation in the cecum by increasing the abundance of *Faecalibacterium* and *Butyricimonas* and ultimately improves the content of butyrate in the cecum. Our gas chromatography results also showed that the butyrate content was significantly increased in G→B rather than GG and BB, but the acetate and propionate concentration in the cecum between G→B, GG and BB had no significant difference. Butyrate, as a modulator of intestinal mucosal immunity, could regulate IL-6 (37) or TNF protein secretion (38), activate B lymphocyte to promote antibody production, and induce T lymphocyte proliferation (39). Interestingly, the KEGG pathway enrichment results based on metabolic pathways also showed that G→B affected Th1, Th2, and Th17 cell differentiation. Therefore, we speculated that butyrate may play an important role in mediating G→B-induced T lymphocyte proliferation and intestinal immune function enhancement.

Our *in vitro* results also found that pretreatment with butyrate effectively promoted cecal tonsil T cell proliferation through the GPR43/HDAC3/p-STAT3/mTOR pathway in chick. In addition, the Mantel correlation analysis also showed that the cecal butyrate concentration was significantly positively correlated with antioxidant enzymes (CAT, GSH-Px, SOD, and T-AOC), anti-inflammation (IL-10), and tight junction protein (ZO-1, claudin-1, and occludin). Thus, we suggested that the elevated level of butyrate content in the cecum is one of the reasons why G→B is a better therapy than GG and BB for promoting T lymphocyte proliferation and improving intestinal immune function. However, in addition to butyrate, other types of intestinal microbiota metabolites also play important roles in mediating the intestinal immune function, such as secondary bile acids (40) and tryptophan (41). Therefore, fecal microbiota transplantation or butyrate supplementation tests should be carried out further in order to verify the core role of butyrate-mediated G→B-induced T lymphocyte proliferation and mucosal immune function enhancement.

## Conclusion

**Figure 12:**
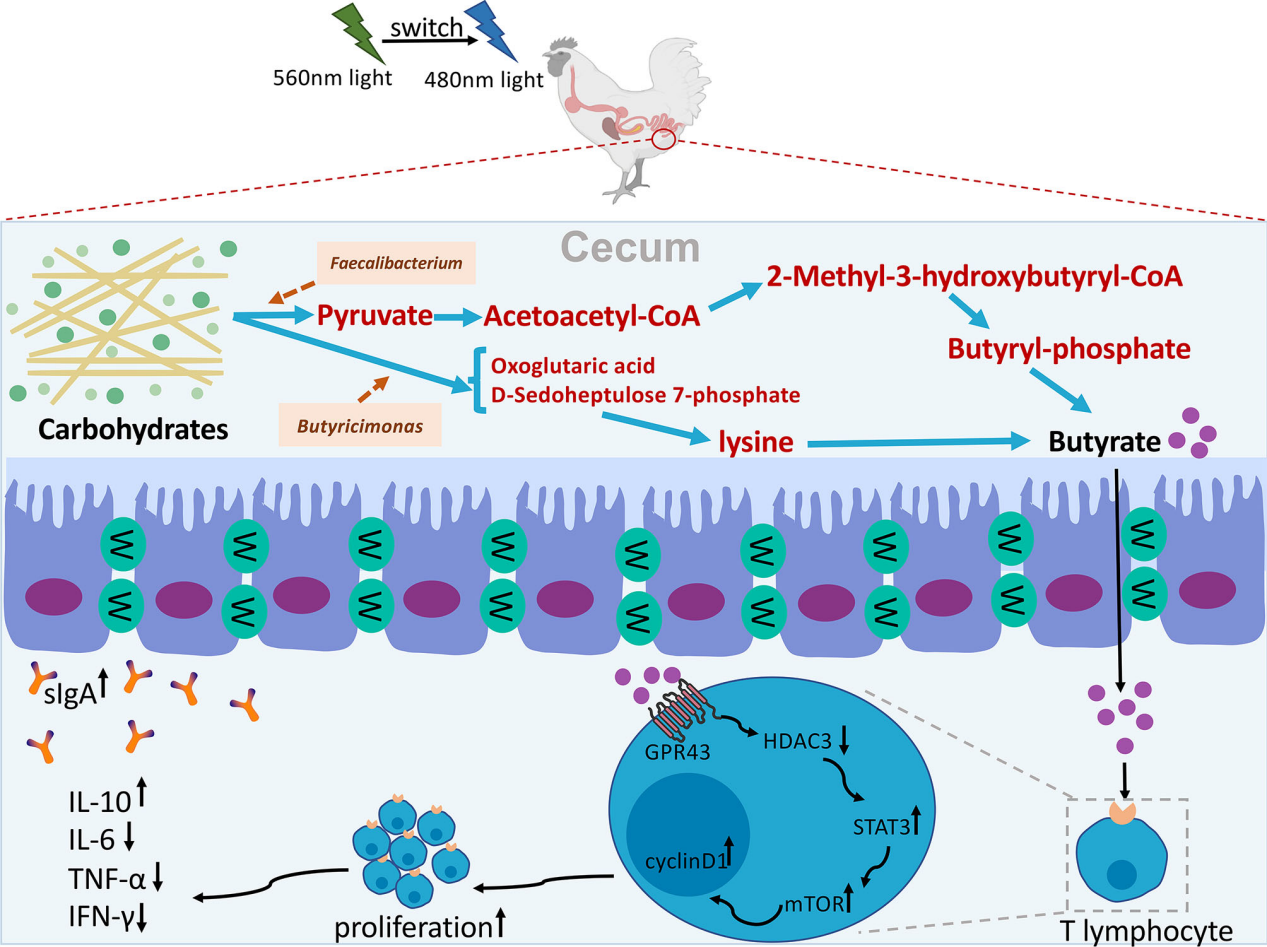
Hypothetical diagram of how G→B mediated cecal microbiome alterations and cecal tonsil T lymphocyte proliferation in chicks. A combination of green and blue monochromatic light indirectly affect the composition of cecal microbiota and increase the relative abundance of Faecalibacterium and Butyricicoccus and butyrate production by reducing the level of oxidative stress in the cecum. Furthermore, butyrate effectively promoted cecal tonsil T cell proliferation through the GPR43/HDAC3/p-STAT3/mTOR pathway in chick.

In summary, our results showed that G→B can indirectly affect the composition of cecal microbiota and increase the relative abundance of *Faecalibacterium* and *Butyricicoccus* and butyrate production by reducing the level of oxidative stress in the cecum. Furthermore, we revealed that pretreatment with butyrate effectively promoted cecal tonsil T cell proliferation through the GPR43/HDAC3/p-STAT3/mTOR pathway in chick. The hypothetical diagram was shown in **Figure 12**.

## Data Availability Statement

The sequencing data we generated were deposited in the NCBI Sequence Read Archive (SRA) under accession numbers from SAMN24603717 to SAMN24603736 in PRJNA739905.

## Ethics Statement

The animal study was reviewed and approved by No. CAU 20171114–2.

## Author Contributions

YC and YZ contributed to the study design. YC obtained funding. YZ performed the experiments. YZ, ZW, JC, and YD analyzed the data. YC and YZ wrote the manuscript. All authors contributed to the article and approved the submitted version.

## Funding

This research was funded by the Chinese National Natural Science Foundation (31873000 and 32172801) and Beijing Natural Science Foundation (grant no. 6222019).

## Conflict of Interest

The authors declare that the research was conducted in the absence of any commercial or financial relationships that could be construed as a potential conflict of interest.

## Publisher’s Note

All claims expressed in this article are solely those of the authors and do not necessarily represent those of their affiliated organizations, or those of the publisher, the editors and the reviewers. Any product that may be evaluated in this article, or claim that may be made by its manufacturer, is not guaranteed or endorsed by the publisher.

## Supplementary Material

Supplementary Figure 1 Kyoto Encyclopedia of Genes and Genomes (KEGG) pathway difference analysis between WW and G→B groups **(A)**. KEGG pathway enrichment analysis between WW and G→B groups **(B)**. WW, white light; RR, red light; GG, green light; BB, blue light; G→B, green light and blue light combination. The results are presented as means ± SEM. Different letters indicate significant differences between the treatments at the same age (*P* < 0.05).

Supplementary Figure 2 Relationships among phenotypic variables, the microbial community, and metabolic butyrate concentration. Pairwise comparisons of phenotypic variables with a color gradient denoting Spearman correlation coefficient. The microbial community and metabolic butyrate concentration were related to each phenotypic variable by Mantel correlation (based on Bray–Curtis dissimilarity). The edge width represents the statistical significance for the corresponding distance correlations, and the edge color denotes Mantel’s *r* statistic. **P* < 0.05; ***P* < 0.01; ****P* < 0.001.
