# Supplementary figures and images for: Effects of Different Monochromatic Light Combinations on Cecal Microbiota Composition and Cecal Tonsil T Lymphocyte Proliferation
Source: Front Immunol. 2022 Jul 12;13:849780. doi: 10.3389/fimmu.2022.849780 (PMC9314779; doi:10.3389/fimmu.2022.849780)

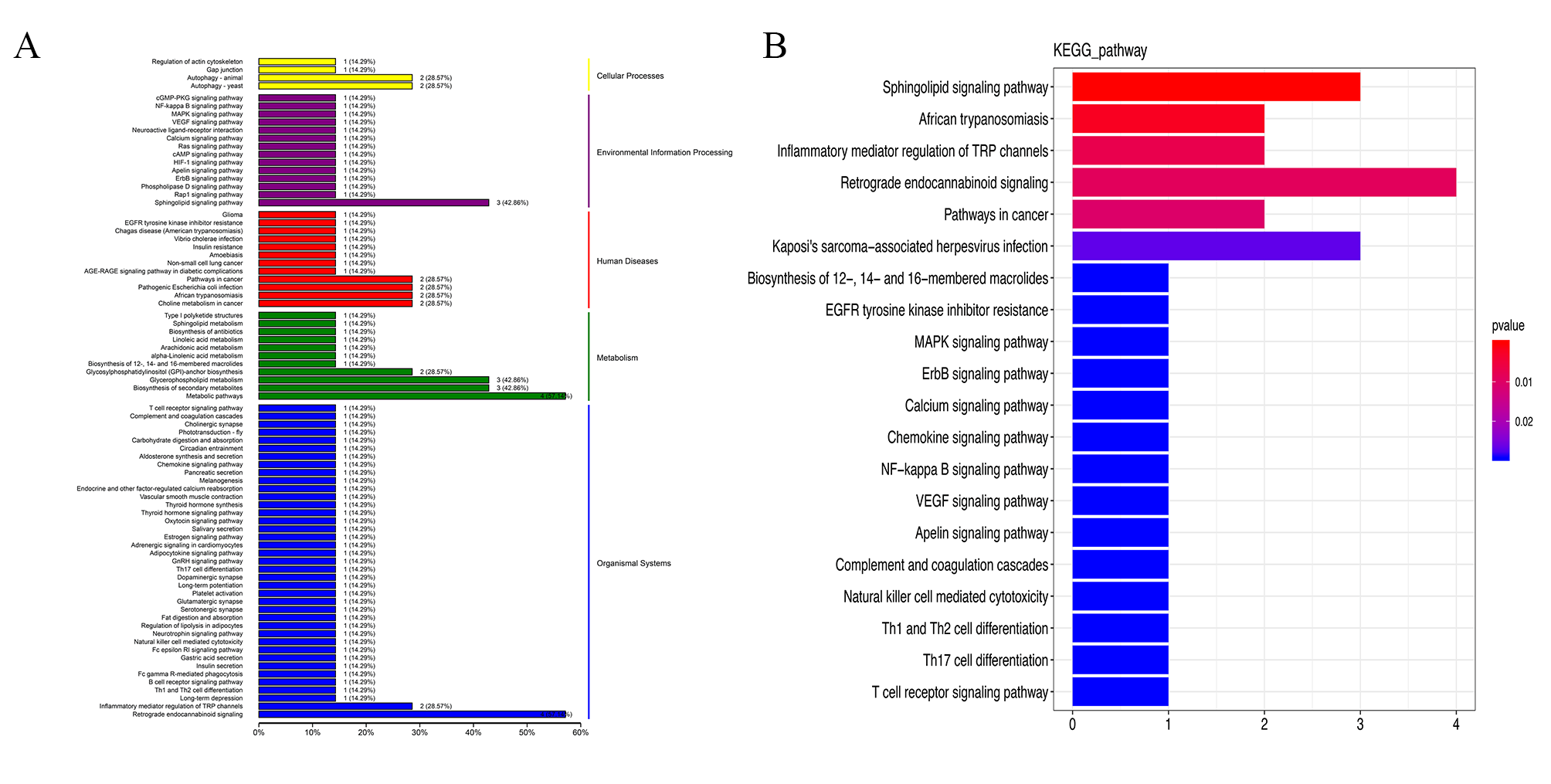

Supplement: Supplementary Figure 1 — Kyoto Encyclopedia of Genes and Genomes (KEGG) pathway difference analysis between WW and G→B groups (A). KEGG pathway enrichment analysis between WW and G→B groups (B). WW, white light; RR, red light; GG, green light; BB, blue light; G→B, green light and blue light combination. The results are presented as means ± SEM. Different letters indicate significant differences between the treatments at the same age (P < 0.05). [file Image_1.tif]

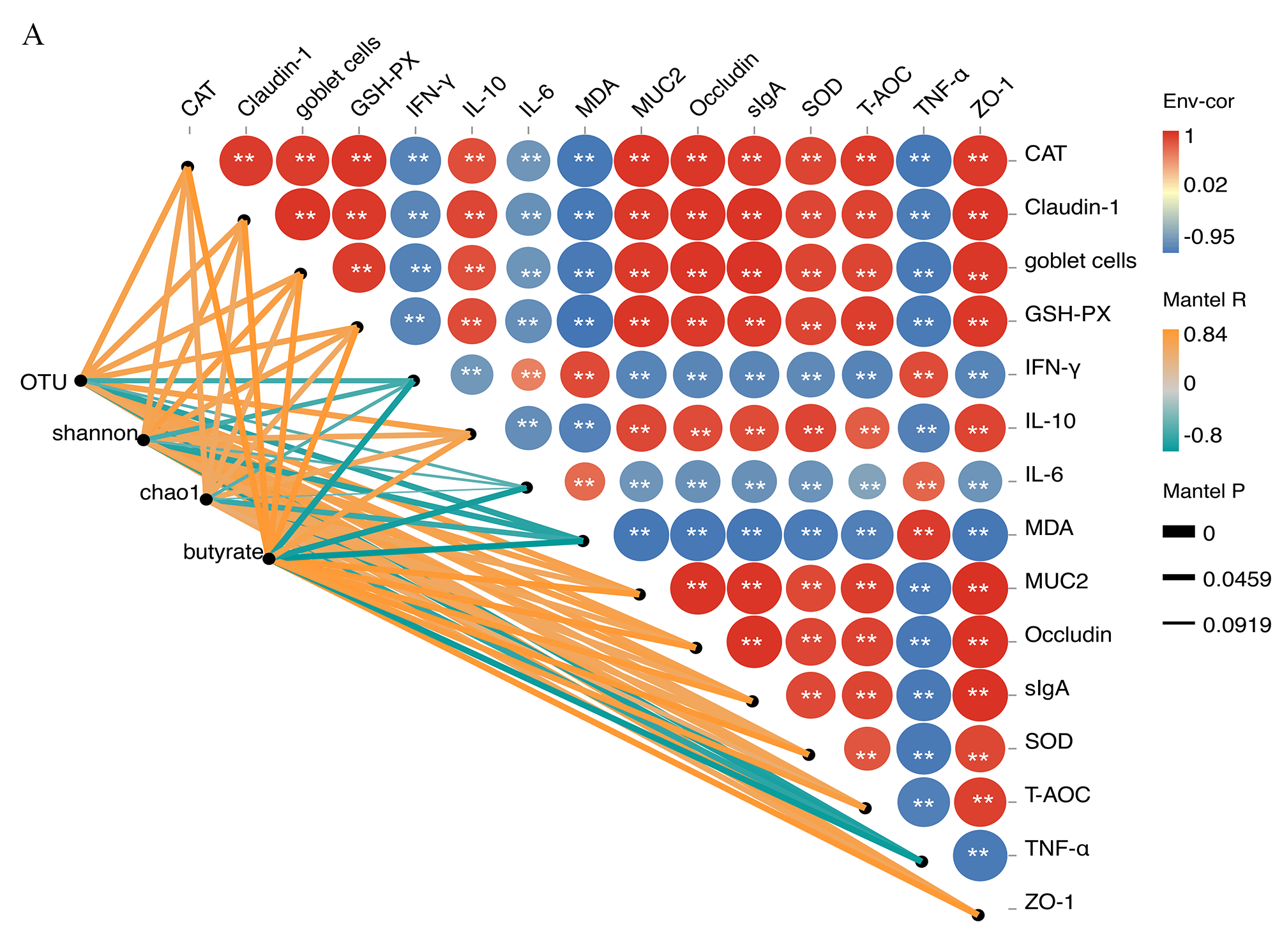

Supplement: Supplementary Figure 2 — Relationships among phenotypic variables, the microbial community, and metabolic butyrate concentration. Pairwise comparisons of phenotypic variables with a color gradient denoting Spearman correlation coefficient. The microbial community and metabolic butyrate concentration were related to each phenotypic variable by Mantel correlation (based on Bray–Curtis dissimilarity). The edge width represents the statistical significance for the corresponding distance correlations, and the edge color denotes Mantel’s r statistic. *P < 0.05; **P < 0.01; ***P < 0.001. [file Image_2.tif]
